# Cardiovascular Disease Risk in Bears and Other Gay Men: A Descriptive Study from Poland

**DOI:** 10.3390/ijerph18031044

**Published:** 2021-01-25

**Authors:** Magdalena Mijas, Karolina Koziara, Andrzej Galbarczyk, Grazyna Jasienska

**Affiliations:** 1 Department of Environmental Health, Faculty of Health Sciences, Jagiellonian University Medical College, 8 Skawinska St., PL 31066 Krakow, Poland; agalbarczyk@gmail.com (A.G.); jasienska@post.harvard.edu (G.J.); 2 Institute of Psychology, Faculty of Philosophy, Jagiellonian University, 6 Ingardena St., PL 30060 Krakow, Poland; karolina.koziara@doctoral.uj.edu.pl

**Keywords:** cardiovascular health, obesity, minority stress, gay men

## Abstract

A risk of cardiovascular disease (CVD) is increased by multiple factors including psychosocial stress and health behaviors. Sexual minority men who identify as Bears form a subculture distinguished by characteristics associated with increased CVD risk such as elevated stress and high body weight. However, none of the previous studies comprehensively investigated CVD risk in this population. Our study compared Bears (*N* = 31) with other gay men (*N* = 105) across a wide range of CVD risk factors. Logistic regression and analysis of covariance (ANCOVA) models were performed to compare both groups concerning behavioral (e.g., physical activity), medical (e.g., self-reported hypertension), and psychosocial (e.g., depressiveness) CVD risk factors. Bears were characterized by older age and higher body mass index (BMI) than the control group. We also observed higher resilience, self-esteem, as well as greater prevalence of self-reported hypertension, diabetes, and hypercholesterolemia in Bears. None of these differences remained statistically significant after adjusting for age and, in the case of self-reported diagnosis of diabetes, both age and BMI. Our study demonstrates that Bears are characterized by increased CVD risk associated predominantly with older age and higher BMI. Health promotion interventions addressed to this community should be tailored to Bears’ subcultural norms and should encourage a healthier lifestyle instead of weight loss.

## 1. Introduction

Cardiovascular disease (CVD) is the leading cause of death worldwide and annually accounts for most deaths associated with noncommunicable diseases [1]. Among Polish men, only in 2014, combined ischemic heart disease, stroke, and other CVDs accounted for as much as 2723 potential years of life lost (PYLL) per every 100,000 men aged 0 to 74 years [2]. It has been estimated that approximately 80% of premature heart disease and stroke is preventable [3].

Risk factors for cardiovascular disease include both behavioral characteristics such as dietary habits, limited physical activity, tobacco use or alcohol consumption, and medical indicators such as high systolic blood pressure, high body mass index, high fasting plasma glucose, and high total cholesterol [2]. Another group of factors that has been studied extensively in the context of CVD is exposure to stress and related mental health adversities such as depressiveness [4]. Chronic stress has been linked in research to vascular pathology (e.g., hypertension), pathogenic changes of the metabolic function (e.g., dyslipidemia), and immune function (e.g., chronic low-grade inflammation), which are the key factors in the development of cardiovascular pathology [5,6]. Psychosocial factors combined with smoking, abnormal lipids, diabetes, hypertension, abdominal obesity, irregular consumption of fruits and vegetables, alcohol intake, and limited physical activity contribute to more than 90% of the CVD risk [4].

Both the relative importance and the distribution of the CVD risk factors vary across different populations, and studies demonstrate that some subgroups may be disproportionately burdened with CVD risk and outcomes compared to others [2,4]. One of such populations characterized by elevated levels of various CVD risk factors is sexual minority men [5]. Compared to heterosexual men, sexual minority men are characterized by higher rates of tobacco use [7,8], excessive alcohol use [9,10], and increased exposure to chronic stress associated with prejudice and discrimination due to minority sexuality [11,12]. A recent systematic review of studies exploring determinants of cardiovascular health in various minority groups demonstrated significant relationships between cardiovascular health indicators and perceived discrimination [13]. Sexual minority men are also disproportionately burdened with health inequalities including a greater prevalence of mental health adversities such as depression and anxiety [14,15], and physical health problems such as cancer or hepatitis [16].

However, the population of sexual minority men is far from homogenous and includes various subgroups and subcultures, which are distinguished by a unique constellation of practices and preferences important for general and cardiovascular health [17,18,19]. One such subculture includes sexual minority men who identify as Bears. Although the Bear subculture originated in the US approximately four decades ago, it has spread all over the world, and organizations of men who identify as Bears exist also in Poland [19]. According to the literature, one in every five sexual minority men may identify as a member of the Bear community [17].

### 1.1. CVD Risk among Bears

Men who identify as Bears are distinguished by their preferences concerning the look of the male body. This includes a larger, heavyset build which combined with more pronounced body and facial hair is regarded as sexually attractive and indicative of masculinity [20,21,22]. The Bear community is more accepting of individuals who do not conform to the mainstream, stereotypical images of gay men—on the contrary, it positively celebrates larger bodies and challenges fat-phobic social norms [23]. A few studies that have been focused on health outcomes and determinants in this population reveal a complex mosaic of factors that potentially shape the health of Bears. They point at both increased and decreased cardiovascular risk among Bears relative to other sexual minority men.

Perhaps the most consistently reported characteristic of Bears, which has been associated in the literature with CVD risk, is increased body weight and related increased body mass index [19,24,25]. Significantly increased body mass index (BMI) values relative to other sexual minority men and/or mean BMI values in a general population indicative of obesity have been observed among both older and younger men from this community (the latter are called Cubs) and Bears living in various sociocultural contexts (i.e., Chinese, Australian, and Polish Bears) [19,24,25]. This disparity is also related to another source of increased CVD risk, which is greater exposure to psychosocial stress. In the case of Bears, greater psychosocial stress, as compared to other sexual minority men, results from exposure to not only sexual minority stigma but also weight stigma, which both may contribute to decreased well-being and increased prevalence of mental health adversities in this population [19,21,23,26]. For example, in a study by Mijas et al. [19], both exposure to weight stigma and sexual minority stigma negatively predicted self-esteem among men who self-identified as Bears.

Interestingly, among Bear-identified men, higher BMI positively predicted self-esteem, which suggests that claiming Bear identity may inspire a change in self-perception and resistance toward dominant cultural body ideals [19]. Qualitative studies also indicate that Bears greatly benefit from becoming a part of an accepting and supporting community, which contributes to increased self-acceptance, enhanced sense of belonging, and strengthened individual resilience [20,26]. This interpretation is supported by studies that failed to observe significant differences between Bears and other sexual minority men concerning depressiveness or social phobia [27] as well as the frequency of being treated for depression or anxiety [25]. Despite being exposed to multiple stigmas, Bears do not seem to suffer from diminished mental health relative to other sexual minority men. Therefore, psychosocial CVD risk factors among Bears compared to other sexual minority men require more research.

Several studies focused on comparing Bears and other sexual minority men concerning such health behaviors as smoking tobacco, alcohol use, and drug use [25,27,28]. Although men from this subculture may be more likely to use illicit substances before or during sexual contacts [27], they do not smoke tobacco more often than other sexual minority men [28] or even smoke less often compared to men from other subcultural groupings within the gay community [25]. Although there is some evidence that social gatherings for Bears are usually associated with increased consumption of alcohol [29], quantitative studies did not demonstrate significantly increased frequencies of excess or binge drinking among Bears [27,28].

None of the previous studies explored quantitatively dietary habits or patterns of physical activity in this population. Only one qualitative study so far has investigated the ways through which Bears navigate their physical activity [23]. Participants in this study struggled with anticipated weight stigma, which caused them to avoid spaces associated with physical activity such as the gym or the beach. This suggests that Bears may be characterized by limited physical activity as compared to other sexual minority men. Similarly, the dietary habits of Bears have been explored by only one study, which was conducted among Brazilian Bears [29]. The study was focused on eating practices among Bears and revealed that certain food preferences (i.e., eating meat and drinking beer) were indicative of dominant subcultural definitions of masculinity and represented a strategy to build the ideal masculine body and avoid discrimination associated with minority sexuality [29]. This study also suggested that eating habits among Bears may increase CVD risk in this population and should be explored in further studies.

Although observed differences between Bears and other sexual minority men suggest that medical CVD risk factors may also disproportionately burden this population, none of the previous studies investigated such indicators of CVD risk among Bears as the prevalence of hypertension, hypercholesterolemia, or diabetes. Our study aimed at filling this gap.

### 1.2. Study Objectives

In this study, we focused on comparing men identified as Bears with other gay men with regard to cardiovascular disease risk factors, including (i) psychosocial characteristics, such as exposure to stigma, self-esteem, resilience, and depressiveness; (ii) behavioral characteristics, such as dietary habits, physical activity, tobacco use, and patterns of alcohol consumption; and (iii) medical indicators, such as diagnoses of hypertension, diabetes, hypercholesterolemia, and body mass index.

### 1.3. Hypothesis

Based on the previous studies, we hypothesized that men who identified as Bears will be characterized by increased cardiovascular risk in case of most medical and behavioral factors, but not psychosocial ones.

## 2. Materials and Methods

### 2.1. Procedure

The study employed data from two research projects. The first one concerned health determinants among members of the Polish Bear community. The invitations for participation in the study were distributed among members and supporters of the Bears of Poland association through social media, mailing lists, and during various social gatherings addressed to members of the community. Participants’ questionnaire data and anthropological measurements were collected during research meetings that had been taking place from June to December 2017 in selected cities in Poland. The study was positively reviewed by the Jagiellonian University Bioethics Committee (122.6120.70.2017). The analyses were performed on the data obtained from 31 cisgender gay men who are members of the Bear community and who incorporated Bear subcultural identification into their identity.

The second research project aimed at examining health determinants within the Polish LGBTQ community (lesbian, gay, bisexual, transgender, and queer community). The study was conducted through the Qualtrics internet platform. The invitations for participation were distributed using the snowball method, through emails, social media, and websites of non-governmental organizations supporting the LGBTQ community. Additional efforts were made to reach members of the LGBTQ community living in smaller towns and villages. The data had been collected between January and March 2018. The study gained a positive review from the Research Ethics Committee of the Institute of Psychology at Jagiellonian University (KE/02/052017). Data from 105 cisgender gay men, who did not identify as Bears, were used in the analyses.

### 2.2. Participants

The analyses employed data obtained from a total of 136 cisgender gay men, including 31 men identifying as Bears and 105 gay men who do not identify as Bears (control group). The average age of the sample was 31.6 years (SD = 10 years). The youngest participant was 18 years old, and the oldest was 69 years old. Most of the participants (*N* = 103; 75.5%) reported a university education level and lived in cities with a population of over 500,000 inhabitants (*N* = 80; 58.8%). One in five participants was experiencing financial hardships (*N* = 26; 19.1%). A comparison of both groups of men in terms of demographic variables is shown in Table 1.

### 2.3. Measures

In both studies, the demographic questionnaire included such information as year of birth, gender and sexual identity, the size of the city of residence, education level, as well as whether the participant’s income is sufficient to cover basic monthly expenses. The study also employed other questionnaire tools, including the Rosenberg Self-Esteem Scale (RSES [30]; in Polish adaptation by Łaguna et al., [31]), the Daily Heterosexist Experiences Questionnaire (DHEQ [32]; in Polish adaptation by Mijas and Koziara [33]), the Resilience Measurement Scale (Skala Pomiaru Prężności SPP-25) [34], the Center for Epidemiologic Studies Depression Scale–Revised (CESD-R [35]; in Polish adaptation by Koziara [36]), and the Dietary Habits and Nutrition Beliefs Questionnaire (KomPAN [37]).

The Rosenberg Self-Esteem Scale (RSES) comprises 10 statements that are to be answered on a four-point scale in terms of how well they reflect the participants’ feelings. The general score of the questionnaire assumes values between 10 and 40 points; the higher scores indicate higher self-esteem. The Polish adaptation of the scale has good psychometric properties and is widely used in research [31].

The Daily Heterosexist Experiences Questionnaire [32,33] was implemented to examine the perceived exposure to the stigma associated with gay identity in surveyed men. The questionnaire consists of 50 items describing various stigmatization experiences, which participants rate using a six-point scale in terms of the degree to which these experiences were stressful for the participants. The response format was as follows: 0 = “Did not happen/not applicable to me”, 1 = “It happened, and it bothered me not at all”, 2 = “It happened, and it bothered me a little bit”, 3 = “It happened, and it bothered me moderately”, 4 = “It happened, and it bothered me quite a bit”, 5 = “It happened, and it bothered me extremely”. The questionnaire covers nine factors, the following six of which were included in the study: Victimization—a factor describing experiences of physical abuse based on a non-heterosexual identity; Harassment and discrimination—a factor including inferior treatment, verbal abuse, and harassment based on sexual identity; Family of origin—a factor depicting experiences of rejection by the family of origin; Vigilance—a factor capturing the effort made to conceal one’s own sexual identity; Isolation—a factor marking feelings of alienation and loneliness experienced as a result of being a non-heterosexual person; and Vicarious trauma—strain resulting from learning about discrimination and abuse experienced by other LGBTQ people. The Polish adaptation of the questionnaire has good psychometric properties, and its general higher scores indicate a higher perceived exposure to stigma [33].

Moreover, the study included the Resilience Measurement Scale SPP-25 [34] to assess the resilience, which is understood as the ability to cope with both daily and traumatic stressors. The scale consists of 25 items to which participants respond using a five-point scale in terms of how accurately the items describe the participants. Higher scores on the scale indicate higher levels of resilience.

The Center for Epidemiologic Studies Depression Scale–Revised (CESD-R) questionnaire [35,36] comprises 20 statements describing a person’s mood and well-being over the last 2 weeks. The study participants rate each statement using a five-point scale in terms of the frequency of symptoms’ or behaviors’ occurrence (where 0 means that the symptom did not occur or lasted no longer than 1 day, and 4 means that it had been occurring almost daily for 2 weeks). Higher scores on the CESD-R scale mean higher intensity of depressive symptoms.

To evaluate positive and negative eating habits, the Dietary Habits and Nutrition Beliefs Questionnaire (KomPAN) was used [37]. It takes into account the frequency of consumption of products from different food groups, which are rated by the participants on a six-point scale, in terms of the participants’ frequency in their consumption (where 1 means “never”, and 6 means “several times a day”). The study included statements necessary to calculate two indices: the healthy diet index (e.g., fruit, vegetables, and fish consumption), and the unhealthy diet index (e.g., fast food, red meat, and sweets consumption).

To assess the prevalence of cardiovascular diseases risk factors, the study employed excerpts from the Behavioral Risk Factor Surveillance System questionnaire (BRFSS [38]) on alcohol consumption, smoking, and physical activity. Questions regarding the frequency of alcohol consumption have been amended so that they would (i) present the volume of different drinks in milliliters, and (ii) refer to the volume of drinks containing ≈10 g of pure ethyl alcohol. To measure the frequency of alcohol consumption, the participants answered the questions of how many days they had drank alcohol drinks during the 30 days preceding the study, and whether during the same period, there had been at least one occasion when they had consumed at least 60 g of pure alcohol (with the drink being defined as 250 mL of 5% beer, 100 mL of 12% wine, or 30 mL of 40% spirits). The participants also answered the question of whether they had ever smoked tobacco and if they smoke now.

To assess physical activity, participants were asked to evaluate how much time (on average) they spend during the week on moderate-intensity physical activity (such as cycling, walking, dancing, or gardening), or vigorous-intensity physical activity (such as jogging, cardio exercises, roller skating, gym exercises, or cross-country skiing). The answers given by the participants have been dichotomized so that they would match the WHO-recommended physical activity for the prevention of chronic diseases, which is at least 150 min of moderate-intensity physical activity or at least 75 min of intense/vigorous physical activity (per week). The dichotomous variable indicated whether or not sufficient physical activity was taking place.

The participants were also to answer the question of whether they had been diagnosed with diabetes, hypertension, or hypercholesterolemia by a medical doctor or other health specialists.

The body height of men identifying as Bears was measured using a portable stadiometer, and their body mass was measured using the Tanita BC-454 Body Composition Monitor. The men in the control group who participated only in the questionnaire study provided information about their body height and current body weight by themselves. Based on the information obtained, the body mass index was calculated for men in both groups.

### 2.4. Data Analysis

The data analysis was performed using the Statistica 13 software (Statsoft, Inc., Tulsa, OK, USA) [39]. Comparisons between the group of Bears and men from the control group in terms of demographic characteristics (such as age, place of residence, education level, or financial hardships), depending on the type of variable, were conducted using an analysis of variance (ANOVA), or logistic regression, to be able to show regression coefficients together with the standard error, or odds ratio together with confidence intervals set at 95%. Comparisons between the groups in terms of the prevalence of health-related behaviors or cardiovascular risk characteristics (i.e., BMI values, patterns of alcohol use, tobacco use, physical activity, as well as mental health and resilience), were conducted while controlling for those demographic variables, with respect to which the groups of men differed significantly (*p* < 0.05). The above analyses were conducted using analysis of covariance (ANCOVA) for quantitative variables, and logistic regression for dichotomous variables. Comparisons of groups of men in terms of the prevalence of medical CVD risk factors (diabetes, hypertension, and elevated cholesterol levels) were performed using logistic regression with adjustment for age and BMI value, which significantly differed men of the two groups. The assumed significance level was 0.05.

## 3. Results

Table 1 presents the comparisons of both groups of men in terms of demographic variables. No statistically significant differences were observed between Bear men and men from the control group regarding the numbers of individuals with university education level, living in cities with a population greater than 500,000, or reporting financial hardships. However, men in the Bear group were on average older than men in the control group (Table 1). As a significant age difference was observed, further comparisons on CVD risk factors included adjustment for age. These comparisons are presented in Table 2.

Men who identify as Bears were found to have significantly higher BMI values, whose mean value was characteristic for obesity (Table 2). This difference remained statistically significant after adjustment for age. No differences between Bear men and men in the control group were observed in terms of depression nor in terms of perceived stigmatization due to sexual identity. However, the men in the Bear group were characterized by both higher resilience and higher self-esteem. These differences were no longer statistically significant after adjustment for age.

In the area of health behaviors (alcohol consumption understood as the number of days during the 30-day period preceding the study when men consumed alcohol and at least one heavy episodic drinking (HED) during the 30 days), no statistically significant differences between the groups were observed. The analogous results were found regarding the percentage of smoking men and the percentage of men undertaking weekly recommended physical activity. Moreover, no statistically significant differences were found in both negative and positive eating habits between the compared groups.

Although men from the Bear group reported a significantly higher prevalence of medical CVD risk factors (diabetes, hypertension, or elevated cholesterol levels), none of these differences remained significant after adjusting models for age and BMI (Table 3).

## 4. Discussion

Our study aimed to compare Bears with other gay men across a range of CVD risk factors including behavioral, medical, and psychosocial ones. Based on previous research, we expected to observe increased levels of medical and behavioral CVD risk factors in Bears compared to other gay men. Consistently with our expectations, Bears were characterized by increased levels of all medical CVD risk factors, including higher BMI, increased frequency of self-reported diagnoses of hypertension, diabetes, and hypercholesterolemia. Of all investigated behavioral CVD risk factors, negative dietary habits index and alcohol consumption were marginally increased in Bears. Among psychosocial characteristics, individual resilience and self-esteem were significantly higher in Bears, indicating better mental health relative to other gay men. None of the observed differences remained statistically significant after adjusting for age and, in the case of self-reported diagnosis of diabetes, both age and BMI.

Increased BMI and older age seem to be the distinguishing characteristics of Bears compared to the gay men from the control group. This result is consistent with previous studies demonstrating that indeed, Bear subculture is particularly welcoming toward older and heavier sexual minority men [25,28,40]. Both characteristics which distinguish members of the Bear subculture have been also consistently and strongly associated with cardiovascular disease [41,42].

Although the body mass index is a convenient tool to monitor the prevalence of obesity at the population level and obesity has been consistently associated with increased CVD morbidity and mortality [41], it has been also demonstrated by numerous studies that obesity defined solely based on the BMI value is a heterogeneous health condition [43]. One of the major contributors to the cardiovascular and metabolic risk associated with increased BMI values and a key factor in obesity heterogeneity is body fat distribution and the tendency to accumulate visceral fat [43]. Another factor largely affecting the CVD prognosis associated with obesity is a physical activity and cardiorespiratory fitness levels [41]. There is some evidence that overweight and obese adults who meet physical activity guidelines (i.e., ≥150 min of moderate-to-vigorous physical activity per week) are characterized by similar or even lower risk for future CVD events as compared to physically inactive adults with normal body weight [44]. This phenomenon is referred to in the literature as a fat-but-fit paradox, and it underscores the importance of promoting physical activity among various populations, including those that are characterized by increased levels of overweight and obesity as well as normal body weight [41].

Aging has also been described in the literature as one of the most important factors affecting cardiovascular homeostasis, since many of the processes that contribute to age-related changes in the vascular structure and function are involved in development of the cardiovascular disease [42]. The prevalence of metabolic syndrome and diabetes is also significantly increased in older populations [42,45]. Therefore, it is not surprising that the observed differences between studied groups concerning being diagnosed with hypertension, diabetes, or hypercholesterolemia were no longer significant after controlling for age, and in case of diabetes, both age and BMI.

We observed similar effects in the case of resilience and self-esteem—significant differences indicating higher self-esteem and resilience in Bears were attributable to age differences between both groups. According to the literature, self-esteem increases during a lifespan and usually peaks between the ages of 60 and 70 years [46]. This result is also consistent with previous studies comparing Bears and other sexual minority men, which indicated significantly decreased self-esteem in Cubs—younger members of the Bear community [25]. It is possible that over the years, Cubs develop strategies to resist discrimination and stigma, which contribute to increased self-esteem in older age. Studies on sexual minority samples demonstrate that throughout their lives, LGBTQ persons indeed develop resilience in the face of experienced adversities [47,48]. Consistent with previous research comparing Bears with other sexual minority men, we also did not observe significant differences between both groups associated with exposure to sexual minority stigma and health outcomes such as depressiveness [25,27]. Given the associations between chronic stigma and cardiovascular health [13], observed exposure to sexual minority stigma in both groups underlines the importance of addressing this issue from a public health perspective. The situation of the LGBTQ community in Poland is particularly difficult including structural inequalities such as no legal recognition of same-sex relationships and recent rise of anti-LGBTQ rhetoric in Polish public discourse. In 2020, Poland has been rated by ILGA-Europe as a country with the worst human rights situation of LGBTQ people among EU Member States [49]. According to data collected by the European Union Agency for Fundamental Rights, of all EU LGBTQ respondents, Polish citizens most often reported being physically or sexually attacked due to their minority identity [50]. This calls for greater involvement of public health experts and policy makers in initiatives aimed at countering the negative impact of stigma on health of LGBTQ persons in Poland.

Among behavioral CVD risk factors, only unhealthy dietary habits and number of drinking days during the 30 days preceding the study were increased in Bears and almost reached the level of statistical significance. However, these disparities may be associated with the way the data were collected and the fact that to reach more participants, we accompanied Bears during their social gatherings, some of which lasted for several days and were associated with increased alcohol consumption and less healthy diet. Consistent with previous studies, we also did not observe significant differences between Bears and other sexual minority men concerning smoking tobacco and excess (heavy episodic) drinking [27,28]. The prevalence of smoking in Bears was similar to corresponding data on the current prevalence of tobacco use in men in Poland which for current daily and occasional smokers was 25.9% [51]. The prevalence of smoking among gay men from the control group was disproportionately higher (Table 2), and as such, it requires more attention from public health experts.

Our study was also the first to compare Bears and other sexual minority men concerning physical activity levels. We did not observe a significant difference between both studied groups in case of reaching physical activity levels recommended by the WHO [52]. Rates of insufficient physical activity in the case of Bears and other gay men were also similar to corresponding rates for Polish men (29% and 25% vs. 31.5% respectively) [53]. Given that an increase from being inactive to reaching recommended physical activity levels is associated with reduced risk of CVD incidence by 17% and diabetes incidence by 26% [54], both Bears and gay men from the control group may benefit from initiatives promoting physical activity.

Our study has several limitations including a small sample size and convenience sampling, which limits the generalizability of the results. We also used self-reported data on CVD risk factors rather than conducting precise measurements. The cross-sectional design of the study limits conclusions on causality. Finally, we compared data from two studies with slightly different methods of data collection (online vs. paper–pencil survey). However, we conducted a study on a very rarely investigated population of Polish sexual minority men, including members of the Polish Bear community who would be extremely difficult to reach through probability sampling methods. Our preliminary observations indicate that future studies should further explore observed effects, preferably using longitudinal research designs and more precise indicators of cardiovascular health such as plasma glucose or low-density lipoprotein cholesterol levels. More studies preferably framed within the intersectionality perspective are also needed on associations between chronic stigma exposure and CVD risk in sexual minority populations. Another important line of research includes intervention studies aimed at testing the effectiveness of cardiovascular health promotion initiatives addressed to both sexual minority men and members of the Bear community.

Our research was also the first to compare Bears and other sexual minority men across such a wide range of CVD risk factors and therefore provides a significant contribution to the literature on intersections of sexualities and health. We demonstrated that certain groups within the broader LGBTQ community may be particularly exposed to CVD health inequalities and that subcultural affiliations of sexual minority men should be included in studies investigating the health of this population. Our analysis has also important practical implications for public health experts and policymakers by indicating the need to further scrutinize observed health disparities.

## 5. Conclusions

Our study demonstrated that men who identify as Bears constitute a distinct population among gay men, which is characterized by increased CVD risk associated predominantly with older age and higher BMI values. Given that more than a quarter of Bears in our sample did not meet the recommended physical activity levels, these differences may contribute to the disproportionate CVD morbidity and mortality in Bears. Health promotion interventions addressed to this community should be tailored to Bears’ subcultural norms, which associates larger body-build with physical attractiveness. Instead of focusing on weight loss, they should encourage a healthier lifestyle, including greater physical activity and healthier dietary habits. Vibrant and close Bear communities make members of this subculture easier to reach and offer an affirmative, non-stigmatizing space to talk about health and healthier life regardless of body size. Public health experts should design their interventions based on these premises.

## Figures and Tables

**Table 1:** A comparison of Bears with other gay men in terms of demographic characteristics.

	**Bear Group (*N* = 31)**		**Control Group (*N* = 105)**		***p***
	**M (SD)**	**β (SE)**	**M (SD)**	**β (SE)**	
Age	37.8 (7.5)		29.8 (9.9)		
		0.34 (0.08)		Ref	<0.001
	**% (N)**	**OR (95% CI)**	**% (N)**	**OR (95% CI)**	***p***
University education	68 (21)		78 (82)		
Unadjusted		0.6 (0.2; 1.4)		Ref	0.240
Adjusted (age)		0.5 (0.2; 1.3)		Ref	0.155
City > 500,000	61.3 (19)		58.1 (61)		
Unadjusted		1.1 (0.5; 2.6)		Ref	0.751
Adjusted (age)		1.0 (0.4; 2.3)		Ref	0.928
Financial hardships (yes)	19.4 (6)		19 (20)		
Unadjusted		1.0 (0.4; 2.8)		Ref	0.970
Adjusted (age)		1.8 (0.6; 5.7)		Ref	0.339

**Table 2:** Comparison of Bears with other gay men in terms of selected behavioral and psychosocial risk factors for cardiovascular diseases.

	**Bear Group (*N* = 31)**		**Control Group (*N* = 105)**		***p***
	**M (SD)**	**β (SE)**	**M (SD)**	**β (SE)**	
BMI	34.7 (6.0)		23.6 (3.5)		
Unadjusted		0.74 (0.06)		Ref	<0.001
Adjusted (Age)		0.65 (0.06)		Ref	<0.001
Sexual minority stigma (DHEQ)	1.42 (0.7)		1.37 (0.6)		
Unadjusted		0.03 (0.09)		Ref	0.721
Adjusted (age)		0.06 (0.09)		Ref	0.477
Resilience (SPP-25)	3.93 (0.5)		3.68 (0.6)		
Unadjusted		0.18 (0.08)		Ref	0.036
Adjusted (age)		0.11 (0.09)		Ref	0.201
Self-esteem (RSES)	32.1 (5.3)		29.5 (6.2)		
Unadjusted		0.18 (0.08)		Ref	0.040
Adjusted (age)		0.10 (0.09)		Ref	0.249
Depression (CESD-R)	16.2 (17.6)		18.0 (16.4)		
Unadjusted		−0.04 (0.09)		Ref	0.608
Adjusted (age)		0.05 (0.09)		Ref	0.591
Alcohol (number of drinking days per 30 days)	8.5 (7.2)		6.1 (6.1)		
Unadjusted		0.15 (0.08)		Ref	0.074
Adjusted (age)		0.12 (0.09)		Ref	0.210
Unhealthy diet index	18.8 (9.5)		15.6 (7.9)		
Unadjusted		0.15 (0.09)		Ref	0.085
Adjusted (age)		0.16 (0.09)		Ref	0.076
Healthy diet index	20 (12.2)		22.3 (12.1)		
Unadjusted		−0.07 (0.09)		Ref	0.393
Adjusted (age)		−0.07 (0.09)		Ref	0.434
	**% (*N*)**	**OR (95% CI)**	**% (*N*)**	**OR (95% CI)**	
Physically active (yes)	71 (22)		76 (63)		
Unadjusted		0.8 (0.3; 1.9)		Ref	0.591
Adjusted (age)		0.8 (0.3; 2.3)		Ref	0.726
Current smoking (yes)	25.8 (8)		41.9 (44)		
Unadjusted		0.5 (0.2; 1.2)		Ref	0.109
Adjusted (age)		0.6 (0.2; 1.5)		Ref	0.282
HED * (≥1 per last 30 days)	62.1 (18)		49 (51)		
Unadjusted		1.7 (0.7; 3.9)		Ref	0.217
Adjusted (age)		1.6 (0.7; 3.9)		Ref	0.298

Note: * HED—heavy episodic drinking.

**Table 3:** Comparison of Bears with other gay men in terms of the prevalence of medical risk factors for cardiovascular diseases.

	Bear Group (*N* = 31)		Control Group (*N* = 105)		*p*
	% (*N*)	OR (95% CI)	% (*N*)	OR (95% CI)	
Hypertension	32.3 (10)		15.5 (16)		
Unadjusted		2.6 (1.0; 6.5)		Ref	0.043
Adjusted (age)		1.8 (0.7; 4.9)		Ref	0.217
Adjusted (age and BMI)		1.5 (0.3; 6.3)		Ref	0.592
Hypercholesterolemia	38.7 (12)		20.2 (21)		
Unadjusted		2.5 (1.0; 5.9)		Ref	0.039
Adjusted (age)		1.5 (0.6; 3.8)		Ref	0.428
Adjusted (age and BMI)		1.4 (0.4; 5.9)		Ref	0.602
Diabetes	13 (4)		1 (1)		
Unadjusted		15.4 (1.6; 143.5)		Ref	0.016
Adjusted (age)		16.4 (1.1; 250)		Ref	0.044
Adjusted (age and BMI)		8.1 (0.3; 227.4)		Ref	0.221

## Data Availability

The data presented in this study are available on request from the corresponding author. The data are not publicly available due to privacy restrictions.
